# SnS_2_ Nanoparticles and Thin Film for Application as an Adsorbent and Photovoltaic Buffer

**DOI:** 10.3390/nano12020282

**Published:** 2022-01-17

**Authors:** Sreedevi Gedi, Salh Alhammadi, Jihyeon Noh, Vasudeva Reddy Minnam Reddy, Hyeonwook Park, Abdelrahman Mohamed Rabie, Jae-Jin Shim, Dohyung Kang, Woo Kyoung Kim

**Affiliations:** 1 School of Chemical Engineering, Yeungnam University, Gyeongsan-si 38541, Korea; drsrvi9@gmail.com (S.G.); salehalhammadi.1987@gmail.com (S.A.); jihyun5946@naver.com (J.N.); drmvasudr9@gmail.com (V.R.M.R.); greatekal@naver.com (H.P.); jjshim@yu.ac.kr (J.-J.S.); 2 Petrochemical Department, Egyptian Petroleum Research Institute, Nasr City, Cairo 11727, Egypt; abdoepri@gmail.com

**Keywords:** nanoparticles, thin films, SnS_2_, dyes, RhB, adsorbent, Cu(In,Ga)Se_2_, solar cell

## Abstract

Energy consumption and environmental pollution are major issues faced by the world. The present study introduces a single solution using SnS_2_ for these two major global problems. SnS_2_ nanoparticles and thin films were explored as an adsorbent to remove organic toxic materials (Rhodamine B (RhB)) from water and an alternative to the toxic cadmium sulfide (CdS) buffer for thin-film solar cells, respectively. Primary characterization tools such as X-ray photoelectron spectroscopy (XPS), Raman, X-ray diffraction (XRD), and UV-Vis-NIR spectroscopy were used to analyze the SnS_2_ nanoparticles and thin films. At a reaction time of 180 min, 0.4 g/L of SnS_2_ nanoparticles showed the highest adsorption capacity of 85% for RhB (10 ppm), indicating that SnS_2_ is an appropriate adsorbent. The fabricated Cu(In,Ga)Se_2_ (CIGS) device with SnS_2_ as a buffer showed a conversion efficiency (~5.1%) close to that (~7.5%) of a device fabricated with the conventional CdS buffer, suggesting that SnS_2_ has potential as an alternative buffer.

## 1. Introduction

Global energy consumption is anticipated to increase dramatically over the next few decades. This is primarily due to the expected global population expansion as well as the economic and industrial growth of developing nations, among other factors. According to a new report from the International Energy Agency (IEA), worldwide energy consumption has declined by approximately 1% in 2020 because of the COVID-19 pandemic and is expected to increase by approximately 5% in the coming years [1]. Sustainable energy sources are growing rapidly, but not fast enough to meet the significant increase in global energy demand, resulting in a dramatic increase in coal consumption that threatens to increase carbon dioxide emissions from the energy industry to historic levels. Electricity generation from PV technology is more affordable than that from non-renewable fossil fuel sources. As a result, it is necessary to advance the field of photovoltaic (PV) research by utilizing sustainable materials.

Notably, solar technologies based on Cu(In, Ga)Se_2_ (CIGS) thin-film semiconducting materials are available at a price comparable to or lower than that of standard silicon modules [2]. The typical structure of CIGS thin film technology comprises a bottom contact layer made of molybdenum (Mo), a p-type CIGS absorber layer (1–3 μm), a thin n-type cadmium sulfide (CdS) buffer layer, a zinc oxide (ZnO)-based transparent window layer [3], and top metal contacts. Generally, conventional CdS buffer is produced by chemical bath deposition (CBD), which shields the absorber layer from direct current (DC) sputtering damage and alters the surface of the CIGS absorber [4]. In addition, the chemical bath eliminates natural oxides from the surface of the CIGS during CdS deposition [5]. CdS can also develop a suitable band alignment with CIGS and transparent conductive oxide [6]. Currently, the world-record efficiency of CIGS solar cells using conventional CdS buffer is approximately 23.35% [7] at the laboratory scale. However, CdS has a few disadvantages. Because of its relatively low band gap (E_g_) (2.4 eV), some of the incident light is absorbed by the CdS buffer (parasitic light absorption), which reduces the available photocurrent (loss in short circuit current of 2 mA/cm^2^) [8]. Further, there is a decrease in the external quantum efficiency (EQE) of CIGS cells with a CdS buffer in the blue region (350–550 nm) owing to the strong recombination of minority carriers (holes) in the window and buffer layers of CIGS cells [5]. However, the primary disadvantage of CdS buffer is its toxicity, which raises concerns on environmental and human health factors [9]. To resolve the disadvantages mentioned above, it is critical to replace the standard CdS buffer layer with one that is environmentally benign, affordable, and provides a significantly higher bandgap compared with CdS.

Another major concern confronting the world is the contamination of water by organic dye solutions that are disposed by the textile, paint, leather, and paper industries [10,11]. Dyes are coloring agents that can be used to tint other materials. Cationic dyes can dissociate into positively charged ions in an aqueous solution, whereas anionic dyes dissociate into negatively charged ions in an aqueous solution. The primary distinction between cationic and anionic dyes is that cationic dyes are basic and anionic dyes are acidic. By combining basic and acidic dyes, neutral dyes are created. Rhodamine B (RhB), methyl orange (MO), and neutral phenol red (PR) are all examples of cationic, anionic, and neutral dyes, respectively [12]. These dyes are highly toxic substances that are harmful to human health, marine life, and the environment [13]. Therefore, dyes must be removed from aqueous solutions prior to disposal. There are different physical [14], chemical [15], and biological [16] decolorization methods that have been used to purify wastewater that contains dyes. Indeed, most dyes are chemically stable for their intended purposes and are challenging to break down biologically [17]. As a result, photocatalysis [18,19], chemical oxidation [20], and adsorption [21] are the primary methods for removing dyes from aqueous solutions. Adsorption is viable for removing hazardous dyes from aqueous solutions as it is simple to conduct, inexpensive, environmentally benign, efficient, and requires little energy [22].

To address the two distinct types of challenges faced by the world, the authors suggested a single material as the solution. Tin disulfide (SnS_2_) is a benign, affordable, and simple binary IV–VI group metal chalcogenide with a band gap of ~2.9 eV and an n-type conductivity. It consists of earth abundant elements with easily controllable chemical stoichiometry and high chemical and environmental stability. It showed a peculiar CdI_2_-type layered structure, with tin atoms sandwiched between two layers of hexagonally arranged close-packed sulfur atoms [23]. It forms an Ohmic contact with metals without current losses. In addition, excellent structural flexibility, broader spectral response, and better thermal stability of SnS_2_ make it a competitive non-toxic substitute for various applications, such as solar cells, field effect transistors, thin film diodes, high-speed photodetectors, lithium-sodium ion batteries, supercapacitors, catalysts, and gas sensors [24,25,26,27,28]. Because of its appropriate band gap, it transmits most of the solar radiation to the absorber and minimizes the parasitic absorption loss. It has carrier density of the order of 10^19^ cm^−3^ and electron affinity of 4.1 eV [29] to achieve sufficient type-inversion within the absorber surface and to make a favorable conduction band offset (CBO, 0–0.4 eV) with the absorber, respectively [30]. Further, it is chemically stable in both acidic and neutral aqueous solutions [21]. Therefore, SnS_2_ has the potential to be a promising non-toxic buffer in solar cells [29,31] and an effective adsorbent for the degradation of organic pollutants [32].

In this paper, we present the synthesis of SnS_2_ nanoparticles using a facile chemical precipitation approach. Subsequently, SnS_2_ thin films were formed by spin coating the SnS_2_ nanoparticles. Further, the SnS_2_ nanoparticles were investigated as a possible adsorbent for removing a toxic dye, Rhodamine B (RhB), from an aqueous solution. In addition, SnS_2_ thin films were explored as a buffer layer for CIGS solar cells.

## 2. Materials and Methods

### 2.1. Reagents

Stannous chloride (SnCl_4_·5H_2_O, AR, purity 99.0%, Sigma-Aldrich, St. Louis, MO, USA) and thioacetamide (C_2_H_5_NS, AR, purity ≥ 99%, Sigma-Aldrich, St. Louis, MO, USA) were used as Sn and S precursors, respectively. Trisodium citrate (Na_3_C_6_H_5_O_7_, AR, purity 99.9%, Sigma-Aldrich, St. Louis, MO, USA) was used as chelating agent. These compounds were utilized as supplied without additional purification and stored in a humidity-controlled desiccator.

### 2.2. Synthesis of SnS_2_ Nanoparticles for Adsorbent Application

**Figure 1:**
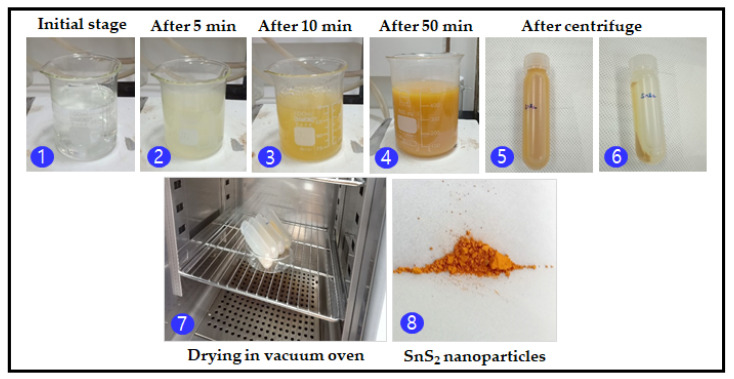
Schematic representation of SnS_2_ nanoparticles synthesis.

The procedure for the synthesis of SnS_2_ nanoparticles is shown in Figure 1. A simple chemical precipitation method was used to synthesize SnS_2_ nanoparticles. For a typical synthesis, 0.1 M of stannous chloride, 0.8 M of thioacetamide, and 1 M of trisodium citrate were aggressively stirred on the magnetic hot plate. The solution was allowed to react for 50 min at a constant temperature of 60 °C with a solution pH of 2. The SnS_2_ nanoparticles were collected and cleaned using deionized water before drying in a vacuum oven at 70 °C for 4 h. Finally, the as-synthesized SnS_2_ nanoparticles exhibited a golden yellow hue. The SnS_2_ nanoparticles/powder was collected in a vial and desiccated for further analyses.

### 2.3. Preparation of SnS_2_ Thin Film for Photovoltaic Application

**Figure 2:**
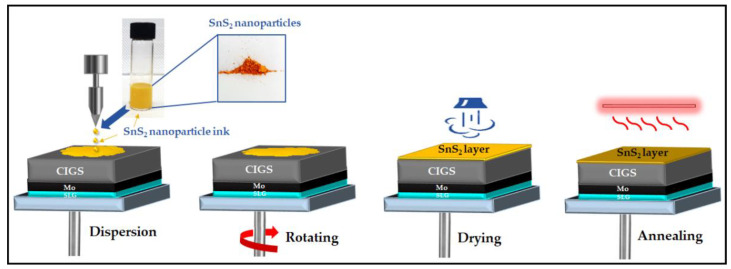
Schematic representation of SnS_2_ thin film preparation.

The as-synthesized SnS_2_ nanoparticles were used for the preparation of SnS_2_ thin films by spin coating. SnS_2_ thin film deposition was accomplished by sonicating the SnS_2_ nanoparticles dispersed in 2 mL ethanol. Then, the resultant ink was spread out over a pre-cleaned glass substrate and a Mo/CIGS absorber. The preparation process of the SnS_2_ thin-film is shown in Figure 2. The SnS_2_ film deposited on a glass substrate was used to analyze the structural and optical properties, and the film on the Mo/CIGS absorber was utilized as a buffer during the construction of a CIGS solar cell for device analysis. The experiments for the synthesis of SnS_2_ nanoparticles and thin films were repeated, and both of the experimental results showed good reproducibility.

### 2.4. Characterization Details

Characterization of SnS_2_ nanoparticles/thin films was done using the following techniques. The phase and elemental purity of the SnS_2_ nanoparticles were analyzed using a Raman spectrometer (Jobin-Yvon Lab Ram HR 800, Horiba, Kyoto, Japan) and X-ray photoelectron spectroscopy (XPS; K-Alpha, Thermo Fisher Scientific, Altrincham, UK), respectively. The structural, morphological and optical properties of the SnS_2_ thin films were studied using a Seifert 3003TT X-ray diffractometer (Almelo, The Netherlands) with CuKα radiation (λ = 1.5405 Å), scanning electron microscope (SEM; Hitachi S-4800, Tokyo, Japan) and UV-Vis-NIR spectrophotometer (Cary 5000, Agilent, Santa Clara, CA, USA), respectively. The changes in the absorbance spectra of RhB in aqueous solution in the presence of SnS_2_ nanoparticles were recorded on a UV-Vis absorption spectrophotometer (Hitachi U-3010, Tokyo, Japan). The photovoltaic performance of the CIGS device with SnS_2_ thin film as a buffer layer was studied using illumination current density, and the voltage (J–V) characteristics were evaluated using a Solar Simulator (McScience K201 LAB50, Suwon, Korea) under AM 1.5 and 100 mW/cm^2^ illumination, where the light intensity was calibrated using an Si standard reference cell.

## 3. Results and Discussion

### 3.1. SnS_2_ Nanoparticles for Adsorbent Application

#### 3.1.1. Growth of SnS_2_ Nanoparticles

The following growth process is hypothesized for the synthesis of SnS_2_ nanoparticles. The chelating agent trisodium citrate complexes the Sn^4+^ ions from stannous chloride in the first stage. The following stage involves the release of S^2−^ ions into the bath as a result of thioacetamide hydrolysis. The precipitation interaction between Sn^4+^ and S^2−^ leads to the production of SnS_2_ nanoparticles in the final stage. The following equations describe the growth stages of the entire process:

SnCl_4_∙5H_2_O + Na_3_C_6_H_5_O_7_ ⟺ Sn (Na_3_C_6_H_5_O_7_)^4+^ + 4Cl^−^ + 5H_2_O

CH_3_CSNH_2_ + H_2_O ⇌ CH_3_CONH_2_ + H_2_S

H_2_S + H_2_O ⇌ H_3_O^+^ + HS^−^

HS^−^ ⇌ H^+^ + S^2−^

HS^−^ + OH^−^ ⇌ H_2_O + S^2−^

Sn^4+^ + S^2−^ → SnS_2_

#### 3.1.2. Phase Purity Confirmation of SnS_2_ Nanoparticles

**Figure 3:**
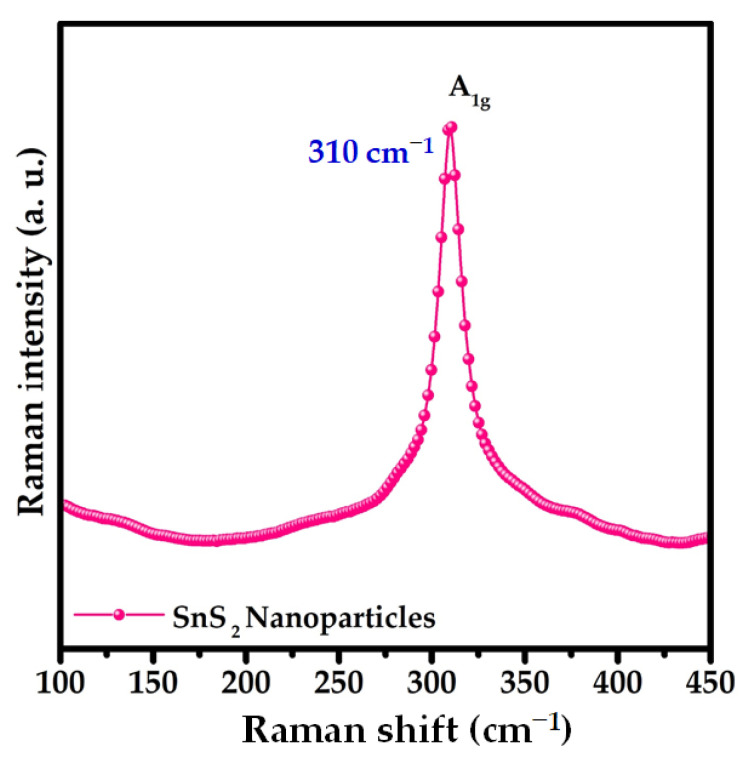
Raman spectrum of SnS_2_ nanoparticles.

The phase purity of the prepared nanoparticles was confirmed using Raman spectroscopy. The Raman spectrum of the as-synthesized nanoparticles in the region of 100–400 cm^−1^ is shown in Figure 3. The spectrum reveals that the as-prepared SnS_2_ nanoparticles exhibited a broad peak at 310 cm^−1^, which corresponds to the Raman mode associated with the hexagonal structure of SnS_2_. The phonon mode at 310 cm^−1^ is attributed to the vertical plane vibration (A_1g_) of the Sn–S bonds [31]. The detected Raman phonon mode was consistent with that of the previous reports [29].

#### 3.1.3. Elemental Purity Confirmation of SnS_2_ Nanoparticles

**Figure 4:**
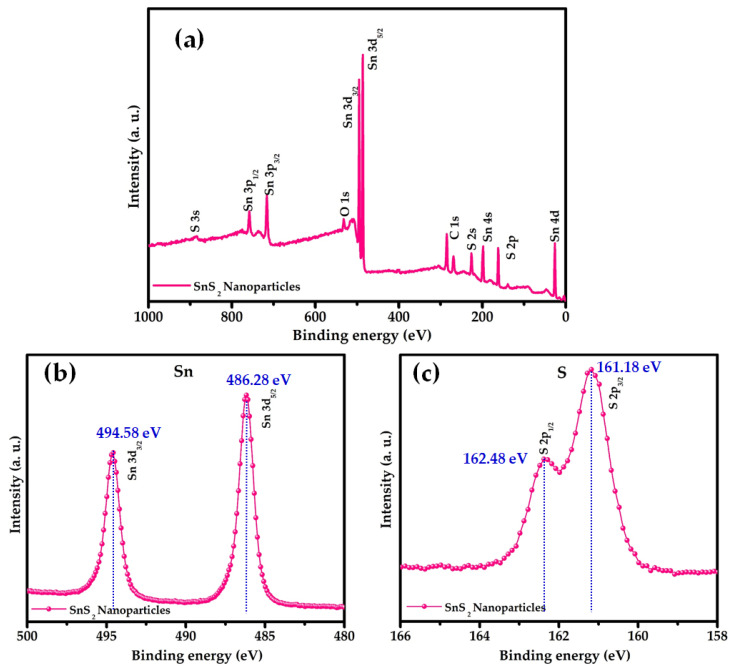
(**a**) Full-scan XPS spectrum and high-resolution scan of the (**b**) Sn 3d core level and (**c**) S 2p core level of SnS_2_ nanoparticles.

XPS was used to characterize the composition of the prepared nanoparticles. No significant peaks representing the contaminants were found in the spectra, suggesting that the impurity level was less than the XPS resolution limit (1 at %). Figure 4a shows a typical high-resolution spectrum of the as-synthesized SnS_2_ nanoparticles with binding energy ranging from 0 to 1000 eV. The wide spectrum revealed numerous peaks, such as S 2s, double S 2p, Sn 3s, doublet Sn 3p, doublet Sn 3d, and Sn 4d, indicating that the synthesized nanoparticles contain Sn and S elements. The peaks linked to C and O were possibly caused by ambient pollution. No other peaks corresponding to other elemental impurities were found in the spectrum. Figure 4b,c show the narrow XPS scans of the Sn 3d and S 2p peaks, respectively. A core-level scan of the Sn 3d doublet revealed two peaks with binding energies of 486.3 eV and 494.6 eV, corresponding to the Sn 3d_5/2_ and Sn 3d_3/2_ energy levels of Sn atoms in the Sn^4+^ valence state, respectively. The peak splitting energy of Sn 3d_5/2_ and Sn 3d_3/2_ levels was approximately 8.3 eV. The binding energies of Sn 3d doublets and their spin energy separations were similar to the values reported [33]. A complete spectrum of the S 2p core level revealed two peaks with binding energies of 162.5 eV and 161.2 eV, which correspond to the S 2p_5/2_ and S 2p_3/2_ energy levels of S atoms in the Sn^2−^ valence state, respectively, which is consistent with the energies of the S^2−^–Sn^4+^ bond, confirming the presence of a single-phase SnS_2_ in the synthesized nanoparticles [34].

#### 3.1.4. SnS_2_ Nanoparticles as Adsorbent for Organic Pollutants (RhB Dye) 

**Figure 5:**
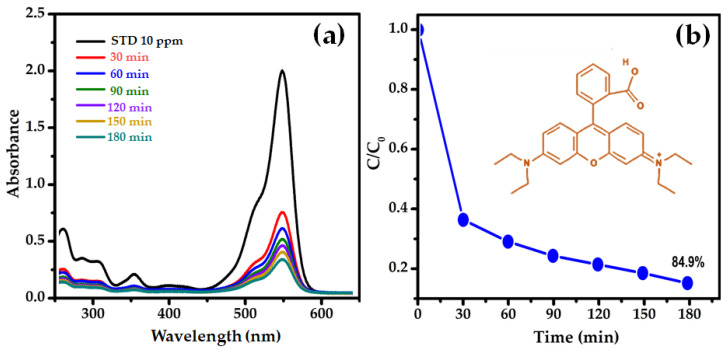
(**a**) Absorbance spectra of the RhB in aqueous solution at different reaction times and (**b**) degradation of the RhB solution over SnS_2_ nanoparticles.

The adsorption of RhB dye from an aqueous solution was carried out at room temperature (approximately 25 °C). Typically, 0.4 g of SnS_2_ nanoparticles was added to a 1 L aqueous solution containing 10 ppm of RhB. The absorbance of RhB was monitored using UV-Vis spectroscopy for 30–180 min. The absorption spectrum of the RhB solution containing SnS_2_ nanoparticles is shown in Figure 5a. The absorbance spectra indicated here were taken for few drops of RhB solution after being centrifuged at a high speed (10,000 RPM for 15 min). So, the SnS_2_ nanoparticles in the sample are resting at the bottom of the sample, meaning there is no peak (at ~427 nm) related to SnS_2_. The intensity of the absorption band at 552 nm decreased linearly with the increase in time, showing that the RhB solution gradually degraded over the SnS_2_ adsorbent. The absorption intensity of the peak decreased by approximately 35% in the presence of SnS_2_ nanoparticles at 30 min. After 60 min, the degree of decolorization was 78%. After 90 min, the degree of decolorization increased to 90%, and then at 180 min, it reached 96%, suggesting that the majority of the RhB had been degraded. Discoloration of the solution might occur as a result of the dye chromogen being destroyed. Similar degradation of RhB with the increasing Zn doping concentration in SnS_2_ nanoparticles was observed in a previous report [35].

The degradation of the RhB solution agrees with the pseudo-first-order kinetics [36,37], ln(C/C_0_) = −kt, where C_0_ and C are the initial and actual concentrations of RhB, k is the rate constant, and t is the degradation time. The normalized concentration of the solution equals the normalized maximum absorbance; therefore, the remaining ratio C/C_0_ is replaced with A/A_0_. The C/C_0_ with respect to reaction time obtained by monitoring the RhB absorption peak at 552 nm is shown in Figure 5b. It is clear that, during the initial 30 min, the concentration of RhB decreased by 65%. Further, the concentration of RhB decreased by 80% and 85% at 120 min and 180 min, respectively. Therefore, within 180 min of reaction time, SnS_2_ nanoparticles showed the highest adsorption capacity (21.25 mg/g) for RhB, indicating that it is an appropriate adsorbent for the removal of RhB.

The adsorption capacity of SnS_2_ nanoparticles was compared to that of other reported nanoparticles and nanocomposites in Table 1, which demonstrated that the adsorption performance of the SnS_2_ nanoparticles synthesized in the present work was similar to that of other nanocomposites and superior to that of other NP adsorbents.

### 3.2. SnS_2_ Thin Films for Photovoltaic Application

**Figure 6:**
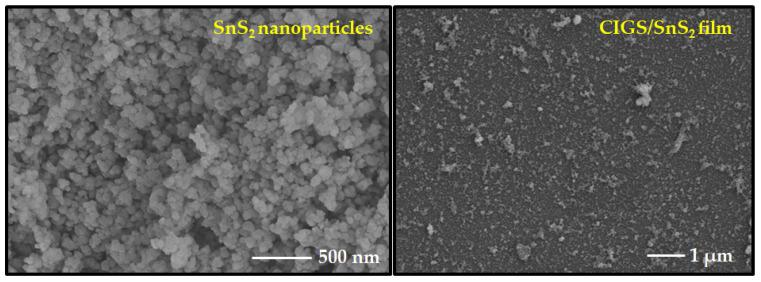
SEM surface image of as-synthesized SnS_2_ nanoparticles and SnS_2_ thin film.

The results obtained in Section 3.1 concluded that the synthesized SnS_2_ nanoparticles have a high degree of composition and phase purity. Therefore, the as-synthesized SnS_2_ nanoparticles were spin coated to obtain the SnS_2_ thin film. The as-synthesized SnS_2_ nanoparticles showed a granular morphology with the particle size of ~52 nm and the as-prepared SnS_2_ thin film on CIGS showed a tiny grain texture with uniformity, as displayed in Figure 6. The structural and optical properties of the SnS_2_ films are described in the following sections.

#### 3.2.1. Crystal Structure of SnS_2_ Thin Film

**Figure 7:**
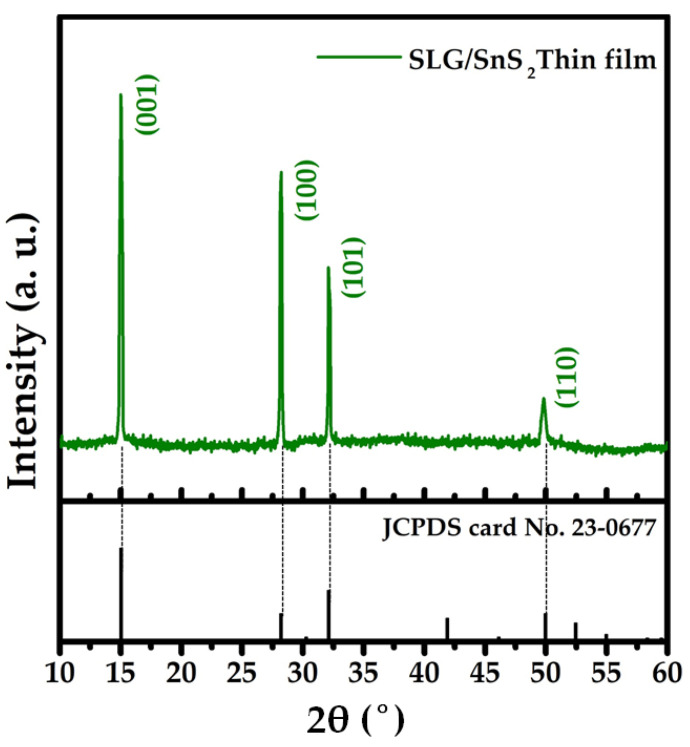
XRD profile of SnS_2_ thin film.

X-ray diffraction was used to determine the crystal structure of the as-grown SnS_2_ film, and the matching profile is shown in Figure 7. The diffraction pattern indicated that the SnS_2_ film was polycrystalline, with significant peaks at 15.02°, 28.18°, 32.54°, and 49.69° corresponding to the (0 0 1), (1 0 0), (1 0 1), and (1 1 0) planes, respectively, and exhibited a hexagonal crystal structure [20]. The SnS_2_ peaks observed in the XRD profile are consistent with those found in the standard JCPDS card No. 23-0677. The film lacked any peaks associated with other secondary phases such as SnS and Sn_2_S_3_, suggesting that the SnS_2_ film generated in this work is free of contaminants [45].

#### 3.2.2. Optical Band Gap of SnS_2_ Thin Film

**Figure 8:**
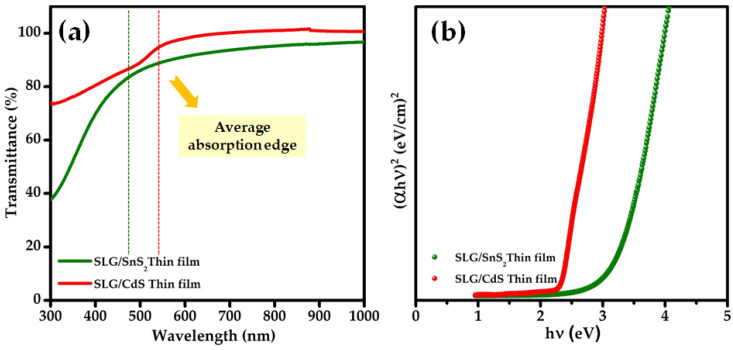
(**a**) Optical transmittance spectrum and (**b**) (αhυ)^2^ vs. hυ plots of SnS_2_ and CdS thin films.

Figure 8a shows the optical transmittance spectrum of the as-grown SnS_2_ film recorded in the wavelength range of 300–1000 nm. A sharp decrease in the transmittance spectrum revealed that the as-grown SnS_2_ film showed an average optical transmittance of approximately 90% above the fundamental absorption edge. The average transmittance difference between CdS and SnS_2_ films in the visible light region is around 11%, which is a small discrepancy that could be attributed to scattering losses. The steep absorption edge in the spectrum indicates that the film has a direct optical transition between the parabolic bands. To evaluate the energy band gap (E_g_) of the thin film, (αhυ)^p^ was plotted against the photon energy (hυ), where p is an integer, whose value indicates the type of optical transition occurring in the thin film. In the present study, the nature of the transition was found to be direct with p = 1/2, and the energy band gap was calculated using plots of (αhυ)^2^ versus hυ (Figure 8b), where the extrapolation of the (αhυ)^2^ plot onto the hυ axis provides the band gap. The evaluated optical energy band gap of the as-prepared SnS_2_ film was approximately 2.8 eV, which is consistent with previously reported findings [23]. Figure 8a,b show the transmittance spectra and corresponding band gap graph of the conventional CdS buffer. Although the transmittance of the standard CdS buffer was similar to that of the SnS_2_ film, the absorption edge of the CdS film was at a longer wavelength than that of the SnS_2_ film. The band gap determined from the (αhυ)^2^ versus hυ plot of CdS was approximately 2.2 eV [46], which is marginally less than the band gap of the SnS_2_ film. In other words, SnS_2_ has a wider band gap than the conventional CdS buffer, which enables the transmission of a more significant fraction of the solar spectrum to the absorber.

#### 3.2.3. SnS_2_ Thin Film as a Buffer in CIGS Solar Cell

In this study, two CIGS thin-film solar cells were fabricated with structures as glass/Mo/CIGS/*CdS* (*conventional buffer*)/i-ZnO/AZO/Ag/Ni and glass/Mo/CIGS/*SnS_2_* (*buffer from the present work*)/i-ZnO/AZO/Ag-Ni using conventional CdS buffer and SnS_2_ buffer, respectively. Our earlier article [47] detailed the procedure for fabricating a CIGS absorber. Initially, a two-stage technique was used to construct a CIGS light absorber on an Mo substrate. Subsequently, the chemical bath deposition (CBD) method produced a 70 nm thick CdS buffer. The complete deposition conditions for CBD-grown CdS are described in a previous report [48]. A 50 nm thick layer of SnS_2_ buffer was spin-coated onto the CIGS absorber following the procedure reported in Section 2.2. The devices were then finished by radio frequency (RF)/DC sputtering of i-ZnO/ZnO/Al transparent conducting layers and e-beam evaporation of an Ni/Al grid.

**Figure 9:**
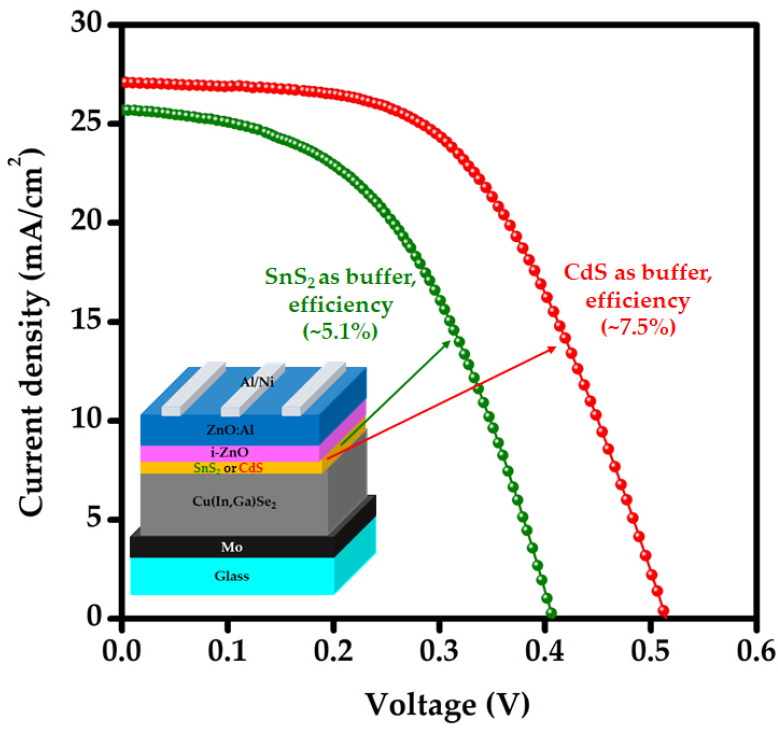
The J–V characteristics of the CIGS devices with conventional CdS and SnS_2_ buffer.

The J–V characteristics of the CIGS devices made with conventional CdS buffer (CIGS_CdS_) and SnS_2_ buffer (CIGS_SnS2_) are shown in Figure 9. The CIGS_CdS_ exhibited an efficiency of 7.5% with an open-circuit voltage (V_OC_) of 0.51 V, a short circuit current density (J_SC_) of 27 mAcm^−2^, and a fill factor (FF) of 54%, while the CIGS_SnS2_ showed an efficiency of 5.1% with a V_OC_ of 0.41 V, J_SC_ of 26 mA cm^−2^, and FF of 49%. The performance parameters of CIGS_SnS2_ and CIGS_CdS_ are summarized in Table 2. The results indicated that the CIGS_SnS2_ device with the SnS_2_ buffer prepared in this study exhibited comparable results to that of the CIGS_CdS_. However, the CIGS_SnS2_ device exhibited a considerable drop in shunt resistance (R_sh_), suggesting the direct contact between the window layer and absorber possibly raised from plasma damage during window layer preparation and shunt paths caused by pinholes in SnS_2_ buffer [49]. As FF is very sensitive to R_sh_ among the device parameters [50], it is reduced by approximately 5% in the case of the CIGS_SnS2_ device. On the other hand, the effect of R_sh_ is less considerable on the J_SC_, which is mainly governed by optical and recombination losses. The optical losses are caused by the reflection from the device surface and absorption by the AZO window and SnS_2_ buffer layers. Electron–hole pairs are generated when photons hit the CIGS absorber, and some of the produced charge carriers are not collected, but are lost as a result of recombination. The recombination loss is indicative of the quality of the SnS_2_/CIGS junction (measure of V_OC_) and is most sensitive to buffer thickness [49]. The plasma damage of SnS_2_ and pinholes may create the AZO/CIGS interface as strong recombination centers. This kind of recombination loss may decrease with the increasing SnS_2_ thickness. According to a previous report [33], the increase in the thickness of the CdS buffer layer resulted in increased light absorption loss by CdS, but decreased reflection loss. Therefore, SnS_2_ thickness optimization is necessary to minimize the photocurrent loss by recombination to ensure the quality of the CIGS/SnS_2_ junction and to improve the performance characteristics of the CIGS_SnS2_ device. 

## 4. Conclusions

Tin disulfide (SnS_2_), a simple binary metal chalcogenide, was proposed as a viable adsorbent for removing toxic dyes from water and as a buffer for Cd-free thin-film solar cells owing to its abundance, low-cost, non-toxicity, and chemical stability. The current study explored the synthesis of SnS_2_ nanoparticles and the deposition of SnS_2_ thin films using a chemical precipitation method and spin-coating technique, respectively. The XPS and Raman studies indicated the existence of Sn and S in the synthesized nanoparticles together with a pure SnS_2_ phase (characteristic Raman mode at 310 cm^−1^). The XRD and optical studies showed that the as-synthesized SnS_2_ thin films had a hexagonal crystal structure with (001) as the preferred orientation and an optical band gap of 2.8 eV. At 180 min of reaction time, 0.4 g/L of SnS_2_ nanoparticles demonstrated 96% decolorization and 85% adsorption capacity for RhB (10 ppm), revealing that the majority of the RhB was degraded; therefore, SnS_2_ is a suitable adsorbent. The fabricated CIGS device with SnS_2_ (50 nm) as buffer exhibited an open circuit voltage (V_OC_) of 0.41 V, a short circuit current density (J_SC_) of 25.67 mA cm^−2^, a fill factor (FF) of 49%, and a conversion efficiency (η) of 5.1%, demonstrating that SnS_2_ is an alternative buffer. The authors intend to dope the nanoparticles and optimize the thickness of the SnS_2_ buffer layer in future studies to (i) increase the adsorption capacity of SnS_2_ nanoparticles and (ii) improve the performance of the CIGS_SnS2_ device.

## Figures and Tables

**Table 1:** Comparison of the adsorption capacity of SnS_2_ nanoparticles with other adsorbent nanoparticles and nanocomposites.

Adsorbent	Adsorption Capacity (mg/g)	Ref.
Fe_3_O_4_/carbon nanocomposite	29.48	[38]
Ni/rGO nanocomposite	65.31	[39]
Fe_3_O_4_/rGO nanocomposite	13.15	[40]
ZnO/rGO nanocomposites	32.6	[41]
CoFe_2_O_4_/MWCNT nanocomposites	35.91	[42]
CoFe_2_O_4_ NPs	5.17	
Fe_3_O_4_/MWCNT nanocomposites	11.44	[43]
ZnFe_2_O_4_ NPs	12.1	[44]
SnS_2_ NPs	21.25	Present work

**Table 2:** Solar cell performance parameters of CIGS devices with conventional CdS and SnS_2_ buffer.

Solar Cell Performance Parameters	Type of Buffer Layer	
	CIGS/CdS	CIGS/SnS_2_
Short circuit current density, J_SC_ (mAcm^−2^)	27.1	25.7
Open-circuit voltage, V_OC_ (V)	0.51	0.41
Fill factor, FF (%)	53.8	49.0
Efficiency, η (%)	7.5	5.1
Shunt resistance, R_Sh_ (Ω cm^2^)	929	255
Series resistance, R_S_ (Ω cm^2^)	14.5	13.2

## Data Availability

Data can be available upon request from the authors.

## References

[1] IEA Global Electricity Demand Is Growing Faster than Renewables, Driving Strong Increase in Generation from Fossil Fuels. https://www.iea.org/news/global-electricity-demand-is-growing-faster-than-renewables-driving-strong-increase-in-generation-from-fossil-fuels.

[2] CIGS-PV-Net CIGS Thin-Film Photovoltaics-News. http://cigs-pv.net/%0Ahttp://files/98/CIGS-WhitePaper.pdf%0Ahttp://files/100/cigs-pv.net.html.

[3] Jung H., Park Y., Gedi S., Reddy V.R.M., Ferblantier G., Kim W.K. (2020). Al-doped zinc stannate films for photovoltaic applications. Korean J. Chem. Eng..

[4] Moon D., Gedi S., Alhammadi S., Minnam Reddy V.R., Kim W.K. (2020). Surface passivation of a Cu(In,Ga)Se_2_ photovoltaic absorber using a thin indium sulfide layer. Appl. Surf. Sci..

[5] Witte W., Spiering S., Hariskos D. (2014). Substitution of the CdS buffer layer in CIGS thin-film solar cells. Vák. Forsch. Prax..

[6] Schock H.-W., Noufi R. (2000). CIGS-based solar cells for the next millennium. Prog. Photovolt. Res. Appl..

[7] Solar Frontier Achieves World Record CIGS Thin-Film Solar Cell Efficiency of 23.35%. https://www.solar-frontier.com/eng/news/2019/0117_press.html.

[8] Orgassa K., Rau U., Nguyen Q., Werner Schock H., Werner J.H. (2002). Role of the CdS buffer layer as an active optical element in Cu(In,Ga)Se_2_ thin-film solar cells. Prog. Photovolt. Res. Appl..

[9] Tötsch W. (1990). Cadmium—Towards a rational use of a toxic element. Environ. Manag..

[10] Han S., Liu K., Hu L., Teng F., Yu P., Zhu Y. (2017). Superior adsorption and regenerable dye adsorbent based on flower-like molybdenum disulfide nanostructure. Sci. Rep..

[11] Liu R., Zhang B., Mei D., Zhang H., Liu J. (2011). Adsorption of methyl violet from aqueous solution by halloysite nanotubes. Desalination.

[12] Dutta S., Gupta B., Srivastava S.K., Gupta A.K. (2021). Recent advances on the removal of dyes from wastewater using various adsorbents: A critical review. Mater. Adv..

[13] Hao O.J., Kim H., Chiang P.-C. (2000). Decolorization of Wastewater. Crit. Rev. Environ. Sci. Technol..

[14] Lei C., Pi M., Jiang C., Cheng B., Yu J. (2017). Synthesis of hierarchical porous zinc oxide (ZnO) microspheres with highly efficient adsorption of Congo red. J. Colloid Interface Sci..

[15] Yuan X., Zhou C., Jin Y., Jing Q., Yang Y., Shen X., Tang Q., Mu Y., Du A.-K. (2016). Facile synthesis of 3D porous thermally exfoliated g-C_3_N_4_ nanosheet with enhanced photocatalytic degradation of organic dye. J. Colloid Interface Sci..

[16] Bulai I.M., Venturino E. (2016). Biodegradation of organic pollutants in a water body. J. Math. Chem..

[17] Arshadi M., Mousavinia F., Amiri M.J., Faraji A.R. (2016). Adsorption of methyl orange and salicylic acid on a nano-transition metal composite: Kinetics, thermodynamic and electrochemical studies. J. Colloid Interface Sci..

[18] Baruah M., Ezung S.L., Supong A., Bhomick P.C., Kumar S., Sinha D. (2021). Synthesis, characterization of novel Fe-doped TiO_2_ activated carbon nanocomposite towards photocatalytic degradation of Congo red, *E. coli*, and *S. aureus*. Korean J. Chem. Eng..

[19] Hu L., Yuan H., Zou L., Chen F., Hu X. (2015). Adsorption and visible light-driven photocatalytic degradation of Rhodamine B in aqueous solutions by Ag@AgBr/SBA-15. Appl. Surf. Sci..

[20] Wan D., Li W., Wang G., Chen K., Lu L., Hu Q. (2015). Adsorption and heterogeneous degradation of rhodamine B on the surface of magnetic bentonite material. Appl. Surf. Sci..

[21] Wang S., Yang B., Liu Y. (2017). Synthesis of a hierarchical SnS_2_ nanostructure for efficient adsorption of Rhodamine B dye. J. Colloid Interface Sci..

[22] Hua M., Zhang S., Pan B., Zhang W., Lv L., Zhang Q. (2012). Heavy metal removal from water/wastewater by nanosized metal oxides: A review. J. Hazard. Mater..

[23] Lindwall G., Shang S.L., Kelly N.R., Anderson T., Liu Z.K. (2016). Thermodynamics of the S–Sn system: Implication for synthesis of earth abundant photovoltaic absorber materials. Sol. Energy.

[24] Tripathi S., Kumar B., Dwivedi D.K. (2021). Numerical simulation of non-toxic In_2_S_3_/SnS_2_ buffer layer to enhance CZTS solar cells efficiency by optimizing device parameters. Optik.

[25] Wang Y., Huang L., Wei Z. (2017). Photoresponsive field-effect transistors based on multilayer SnS_2_ nanosheets. J. Semicond..

[26] Sánchez-Juárez A., Tiburcio-Silver A., Ortiz A. (2005). Fabrication of SnS_2_/SnS heterojunction thin film diodes by plasma-enhanced chemical vapor deposition. Thin Solid Films.

[27] Sun Y., Cheng H., Gao S., Sun Z., Liu Q., Leu Q., Lei F., Yao T., He J., Wei S. (2012). Freestanding tin disulfide single-layers realizing efficient visible-light water splitting. Angew. Chem. Int. Ed..

[28] Ou J.Z., Ge W., Carey B., Daeneke T., Rotbart A., Shan W., Wang Y., Fu Z., Chrimes A.F., Wlodarski W. (2015). Physisorption-based charge transfer in two-dimensional SnS_2_ for selective and reversible NO_2_ gas sensing. ACS Nano.

[29] Gedi S., Minna Reddy V.R., Pejjai B., Jeon C.-W., Park C., Ramakrishna Reddy K.T. (2016). A facile inexpensive route for SnS thin film solar cells with SnS_2_ buffer. Appl. Surf. Sci..

[30] Minnam Reddy V.R., Lindwall G., Pejjai B., Gedi S., Kotte T.R.R., Sugiyama M., Liu Z.K., Park C. (2018). α-SnSe thin film solar cells produced by selenization of magnetron sputtered tin precursors. Sol. Energy Mater. Sol. Cells.

[31] Gedi S., Minnam Reddy V.R., Pejjai B., Park C., Jeon C.W., Kotte T.R.R. (2017). Studies on chemical bath deposited SnS_2_ films for Cd-free thin film solar cells. Ceram. Int..

[32] Wu Z., Xue Y., Zhang Y., Li J., Chen T. (2015). SnS_2_ nanosheet-based microstructures with high adsorption capabilities and visible light photocatalytic activities. RSC Adv..

[33] Matyszczak G., Fidler A., Polesiak E., Sobieska M., Morawiec K., Zajkowska W., Lawniczak-Jablonska K., Kuzmiuk P. (2020). Application of sonochemically synthesized SnS and SnS_2_ in the electro-Fenton process: Kinetics and enhanced decolorization. Ultrason. Sonochemistry.

[34] Crist B.V., Crisst D.B.V. (2000). Handbook of Monochromatic XPS Spectra.

[35] Lather R., Jeevanandam P. (2022). Synthesis of Zn^2+^ doped SnS_2_ nanoparticles using a novel thermal decomposition approach and their application as adsorbent. J. Alloy. Compd..

[36] Bayati M.R., Golestani-Fard F., Moshfegh A.Z. (2010). Visible photodecomposition of methylene blue over micro arc oxidized WO_3_–loaded TiO_2_ nano-porous layers. Appl. Catal. A Gen..

[37] Du W., Deng D., Han Z., Xiao W., Bian C., Qian X. (2011). Hexagonal tin disulfide nanoplatelets: A new photocatalyst driven by solar light. CrystEngComm.

[38] Singh K.P., Gupta S., Singh A.K., Sinha S. (2010). Experimental design and response surface modeling for optimization of Rhodamine B removal from water by magnetic nanocomposite. Chem. Eng. J..

[39] Jinendra U., Bilehal D., Nagabhushana B.M., Kumar A.P. (2021). Adsorptive removal of Rhodamine B dye from aqueous solution by using graphene–based nickel nanocomposite. Heliyon.

[40] Sun H., Cao L., Lu L. (2011). Magnetite/reduced graphene oxide nanocomposites: One step solvothermal synthesis and use as a novel platform for removal of dye pollutants. Nano Res..

[41] Wang J., Tsuzuki T., Tang B., Hou X., Sun L., Wang X. (2012). Reduced graphene oxide/ZnO composite: Reusable adsorbent for pollutant management. ACS Appl. Mater. Interfaces.

[42] Oyetade O.A., Nyamori V.O., Martincigh B.S., Jonnalagadda S.B. (2015). Effectiveness of carbon nanotube–cobalt ferrite nanocomposites for the adsorption of rhodamine B from aqueous solutions. RSC Adv..

[43] Kerkez Ö., Bayazit Ş.S. (2014). Magnetite decorated multi-walled carbon nanotubes for removal of toxic dyes from aqueous solutions. J. Nanoparticle Res..

[44] Konicki W., Siber D., Narkiewicz U. (2017). Removal of Rhodamine B from aqueous solution by ZnFe_2_O_4_ nanocomposite with magnetic separation performance. Pol. J. Chem. Technol..

[45] Jeong D., Reddy V.R.M., Pallavolu M.R., Cho H., Park C. (2020). Investigation on the performance of SnS solar cells grown by sputtering and effusion cell evaporation. Korean J. Chem. Eng..

[46] Alhammadi S., Jung H., Kwon S., Park H., Shim J.-J., Cho M.H., Lee M., Kim J.S., Kim W.K. (2018). Effect of Gallium doping on CdS thin film properties and corresponding Cu(InGa)Se_2_/CdS:Ga solar cell performance. Thin Solid Films.

[47] Park Y., Ferblantier G., Slaoui A., Dinia A., Park H., Alhammadi S., Kim W.K. (2020). Yb-doped zinc tin oxide thin film and its application to Cu(InGa)Se_2_ solar cells. J. Alloys Compd..

[48] Alhammadi S., Moon K., Park H., Kim W.K. (2017). Effect of different cadmium salts on the properties of chemical-bath-deposited CdS thin films and Cu(InGa)Se_2_ solar cells. Thin Solid Films.

[49] Cho K.S., Jang J., Park J.H., Lee D.-K., Song S., Kim K., Eo Y.-J., Yun J.H., Gwak J., Chung C.-H. (2020). Optimal CdS buffer thickness to form high-quality CdS/Cu(In,Ga)Se_2_ junctions in solar cells without plasma damage and shunt paths. ACS Omega.

[50] Ghorpade U., Suryawanshi M., Shin S.W., Gurav K., Patil P., Pawar S., Hong C.W., Kim J.H., Kolekar S. (2014). Towards environmentally benign approaches for the synthesis of CZTSSe nanocrystals by a hot injection method: A status review. Chem. Commun..

